# Parental Acceptance of Nirsevimab for RSV Prevention in Infants Across Two Consecutive Seasons in Emilia-Romagna, Italy

**DOI:** 10.3390/vaccines14080696

**Published:** 2026-08-12

**Authors:** Susanna Esposito, Valentina Fainardi, Maria Elena Capra, Melodie O. Aricò, Angela Lanzoni, Francesco Accomando, Gaia Giorgia Arnesano, Cosimo Neglia, Enrico Valletta, Giacomo Biasucci, Serafina Perrone

**Affiliations:** 1 Pediatric Clinic, University Hospital of Parma, 43126 Parma, Italy; valentina.fainardi@unipr.it (V.F.); gaiagiorgia.arnesano@unipr.it (G.G.A.); negliamino@gmail.com (C.N.); 2 Pediatrics and Neonatology Unit, Department of Medicine and Surgery, Guglielmo da Saliceto Hospital, University of Parma, 29100 Piacenza, Italy; m.capra@ausl.pc.it (M.E.C.); giacomo.biasucci@unipr.it (G.B.); 3 Pediatric Unit, G.B. Morgagni-L. Pierantoni Hospital, AUSL Romagna, 47100 Forlì, Italy; melodieolivialoredanarosa.arico@auslromagna.it (M.O.A.); francesco.accomando@auslromagna.it (F.A.); enrico.valletta@auslromagna.it (E.V.); 4 Pediatric Unit, Santa Maria della Scaletta Hospital, 40026 Imola, Italy; angela.lanzoni@ausl.imola.bo.it; 5 Neonatology Unit, University Hospital of Parma, 43126 Parma, Italy; serafina.perrone@unipr.it

**Keywords:** respiratory syncytial virus, RSV, nirsevimab, monoclonal antibody, parental acceptance, infant immunoprophylaxis

## Abstract

**Background**: Respiratory syncytial virus (RSV) is a leading cause of lower respiratory tract infection and hospitalization in infants. Nirsevimab, a long-acting monoclonal antibody, provides single-dose protection during the RSV season, but the effectiveness of prophylaxis programs depends on sustained parental acceptance and high uptake. This study evaluated changes in parental knowledge, perceptions, and willingness to accept nirsevimab across two consecutive RSV seasons in Emilia-Romagna, Italy. **Methods**: Two multicenter cross-sectional surveys were conducted during consecutive RSV seasons, 2024–2025 and 2025–2026, using a comparable questionnaire and recruitment approach. Parents or legal guardians of infants eligible for nirsevimab completed a semi-structured questionnaire during routine counseling in neonatal units. Survey 1 included 1042 respondents and survey 2 included 867 respondents. Sociodemographic characteristics, RSV awareness, knowledge and perception of nirsevimab, willingness to accept prophylaxis, trust in healthcare providers and the healthcare system, preferred information sources, and willingness to pay were compared between seasons. **Results**: Willingness to administer nirsevimab remained high and stable—87.04% in survey 1 and 88.00% in survey 2. Awareness of RSV-related risks increased from 68.23% to 73.47% (*p* < 0.05), and correct identification of nirsevimab as an antibody increased from 65.93% to 71.74% (*p* < 0.01). Explicit refusal rose slightly from 2.21% to 3.81% (*p* < 0.05). In survey 2, acceptance was associated with higher education, awareness of RSV risks, perceived child susceptibility, confidence in efficacy, lower concern about side effects, trust in pediatricians and the healthcare system, and willingness to pay. Notably, acceptance in 2025–2026 was higher among infants born in September–December than among those born in January–March, indicating a late-season decline. **Conclusions**: Parental acceptance of nirsevimab remained high across two seasons. Future campaigns should address residual knowledge gaps, reinforce communication on safety and efficacy, and sustain high coverage throughout the entire RSV season, particularly among infants born in its final months.

## 1. Introduction

Respiratory syncytial virus (RSV) is one of the leading causes of acute lower respiratory tract infections in infants and young children worldwide, and it represents a major cause of bronchiolitis, pneumonia, hospitalization, and, in the most severe cases, admission to intensive care units [1,2]. Although severe RSV disease has traditionally been associated with prematurity, congenital heart disease, chronic lung disease, and other underlying conditions, most RSV-related hospitalizations occur in otherwise healthy term infants because of the large size of this population [2,3]. RSV therefore represents not only a clinical challenge for pediatric and neonatal services, but also a significant public health burden during seasonal epidemics.

In recent years, RSV prevention strategies have changed substantially. For decades, passive immunoprophylaxis was mainly limited to selected high-risk infants through palivizumab, which requires repeated monthly administrations during the RSV season [4]. The development of nirsevimab, a long-acting monoclonal antibody targeting the RSV fusion protein, has expanded the possibility of protecting a broader infant population with a single intramuscular dose administered before or during the RSV season [5]. Nirsevimab has been authorized in Europe for the prevention of RSV lower respiratory tract disease in newborns and infants during their first RSV season [5,6]. Clinical trials and real-world studies have shown that nirsevimab is effective in reducing medically attended RSV disease, RSV-related hospitalizations, and severe outcomes in newborns and infants [7,8,9,10].

Following these data, several countries and regions have progressively introduced nirsevimab into seasonal RSV prevention programs. In Italy, implementation has initially occurred at regional level, with differences in timing, eligibility criteria, and organization of prophylaxis campaigns [11,12]. In Emilia-Romagna, nirsevimab has been implemented as part of a publicly funded regional RSV prevention program and is offered to eligible infants on a voluntary basis without direct cost to families. During the 2024–2025 season, prophylaxis was initially offered to infants born during the epidemic period and to selected infants at increased risk of severe RSV disease, starting in November. For the 2025–2026 season, the campaign was anticipated to September and eligibility was expanded to all infants, while access to nirsevimab remained free of charge for eligible families [13]. In this context, understanding parental knowledge, perceptions, and willingness to accept nirsevimab is essential, because the success of infant RSV prevention programs depends not only on product availability and clinical effectiveness, but also on timely parental acceptance.

Parental decision-making regarding new preventive interventions is complex and may be influenced by awareness of the disease, perceived susceptibility of the child, perceived safety and efficacy of the intervention, trust in healthcare professionals, trust in the healthcare system, and economic considerations [14,15,16,17]. These aspects are particularly relevant for nirsevimab, because it is a relatively new intervention and may be misunderstood by parents as a vaccine rather than as a monoclonal antibody. Clear communication from trusted healthcare professionals, especially pediatricians and neonatologists, may therefore play a crucial role in supporting informed parental choices.

A previous survey conducted in Emilia-Romagna during the 2024–2025 season showed high parental willingness to administer nirsevimab, while also identifying relevant knowledge gaps and concerns about safety [18]. The present manuscript updates those findings by comparing parental responses collected during two consecutive RSV seasons, 2024–2025 and 2025–2026. Specifically, this study aims to evaluate changes over time in parental awareness of RSV, knowledge and perceptions of nirsevimab, willingness to administer nirsevimab to their child, trust in healthcare providers and the national healthcare system, and factors associated with acceptance or hesitancy. By identifying changes between the first and second seasons of implementation, these data may help guide future communication strategies and support more effective and equitable RSV prevention programs. The present study incorporates previously published 2024–2025 data exclusively as a historical comparator for a new, independently recruited cohort surveyed during the 2025–2026 RSV season. Thus, the data collected in Survey 2, the direct comparisons between the two consecutive seasons, and the evaluation of changes in parental attitudes and acceptance over time constitute the new contribution of the present study.

## 2. Methods

### 2.1. Study Design and Population

This study consisted of two multicenter cross-sectional surveys conducted during two consecutive RSV seasons in neonatology and maternity units in the Emilia-Romagna region, Italy. The surveys involved independent samples of parents or legal guardians; individual participants were not followed longitudinally between seasons. The first cross-sectional survey was conducted during the 2024–2025 RSV season and has been previously published [18]. The second survey was conducted during the 2025–2026 season using a comparable methodology. The present study was designed to compare the two independent cross-sectional samples and evaluate changes in parental knowledge, perceptions, and willingness to accept nirsevimab between consecutive seasons.

Survey 1 corresponds to the cohort of 1042 respondents previously described by Esposito et al. [18]; these data were not newly collected for the present manuscript. They were included in the current analysis to permit direct comparison with the independently recruited Survey 2 cohort. Survey 2 data and all between-season comparisons are original to the present study.

Participants were recruited using a consecutive sampling strategy. During the predefined recruitment periods, parents or legal guardians of eligible neonates or infants attending the participating neonatology or maternity units were consecutively invited to participate during routine counseling regarding RSV prevention and nirsevimab administration. Inclusion criteria were: (1) being a parent or legal guardian of a neonate or infant eligible for nirsevimab according to the Emilia-Romagna regional recommendations applicable during the corresponding RSV season; (2) attendance at one of the participating centers during the recruitment period; and (3) provision of written informed consent. Parents or guardians who declined participation or did not provide informed consent were excluded. Participants unable to complete the questionnaire in one of the available languages were also excluded. Only one questionnaire per family/child was included in the analysis, including questionnaires completed jointly by both parents. Eligibility was defined according to the regional recommendations in force during each season. Consequently, the target populations were not completely identical, because the nirsevimab campaign began earlier and was expanded during the 2025–2026 season.

A total of 1042 respondents completed the questionnaire in survey 1, corresponding to the 2024–2025 season, and 867 respondents completed the questionnaire in survey 2, corresponding to the 2025–2026 season. The same general approach used in the previous publication was maintained to allow comparison between the two survey periods. The updated manuscript compares the two cohorts in terms of sociodemographic characteristics, RSV awareness, knowledge and perception of nirsevimab, willingness to administer nirsevimab to the child, trust in healthcare providers and the national healthcare system, and willingness to pay for prophylaxis if required.

The study was approved by the Ethic Committee of the participating centers; participation required that parents or legal guardians provide written informed consent after being fully informed about the study’s objectives and procedures.

### 2.2. Questionnaire

Data were collected using a semi-structured questionnaire composed predominantly of closed-ended, multiple-choice questions. Some items allowed more than one response, and selected questions included an “Other” option permitting respondents to specify an answer not represented by the predefined categories. No fully open-ended questions were included. Respondents could leave individual items unanswered. The same core questionnaire was used in both seasons. Before Survey 2, only minor adaptations were introduced, consisting of updated wording regarding the regional campaign. All items included in the direct comparison between the two surveys retained the same wording and response categories. The questionnaire was designed to explore parental knowledge, attitudes, and perceptions regarding RSV infection and nirsevimab prophylaxis.

The questionnaire development and pilot-testing procedures have been described previously [18]. Briefly, questionnaire development was informed by a review of the literature on parental knowledge and perceptions of RSV, immunization decision-making, and acceptance of new preventive interventions. The content was subsequently reviewed by experts in neonatology, pediatrics, epidemiology, and psychology to assess its relevance, clarity, and consistency with the objectives of the study. Before full implementation, the questionnaire was pilot-tested in 30 parents representative of the target population, including individuals with different educational levels, linguistic backgrounds, and health-literacy profiles, as well as parents of both high- and low-risk infants. Feedback obtained during pilot testing was used to improve the clarity, comprehensibility, relevance, and cultural appropriateness of individual items. The questionnaire was prepared in Italian and translated into English and Arabic, with back-translation used for the non-Italian versions to verify linguistic consistency [18].

The questionnaire included sections on: parental sociodemographic characteristics; number of children in the family; presence of neonatal comorbidities; awareness of RSV and perceived risk of RSV infection; knowledge of RSV-related complications; previous awareness of nirsevimab and sources of information; understanding of nirsevimab as a preventive intervention; willingness to have nirsevimab administered to the child; factors influencing the decision; perceived efficacy and safety of nirsevimab; concern about potential side effects; trust in pediatricians and in the national healthcare system; preferred sources of information; and willingness to pay if nirsevimab were not publicly funded. Because nirsevimab was provided free of charge within the regional program, the question concerning willingness to pay represented a hypothetical scenario intended to explore the potential influence of out-of-pocket cost on parental decision-making.

As in the previous publication, the questionnaire was administered in the context of routine pre-immunization counseling. Its purpose was not to test technical knowledge, but to capture parental perceptions and decision-making factors in a real-world setting during the implementation of the regional nirsevimab prophylaxis campaign.

### 2.3. Outcomes

The primary outcome was parental willingness to have nirsevimab administered to their child, compared between survey 1 and survey 2. Responses were categorized as “yes”, “no”, or “not sure”. For analyses exploring determinants of acceptance, respondents who answered “no” or “not sure” were grouped together and compared with those who answered “yes”.

Secondary outcomes included changes between the two seasons in awareness of RSV-related risks, perceived risk of RSV infection, knowledge of RSV complications, previous awareness of nirsevimab, sources of information, understanding of nirsevimab, perceived efficacy, concern about side effects, trust in healthcare professionals and in the healthcare system, interest in receiving further information, preferred communication channels, and willingness to pay for prophylaxis.

An additional analysis evaluated willingness to receive nirsevimab according to the neonate’s month of birth, comparing neonates born in September–December with those born in January–March in each season and between seasons.

### 2.4. Statistical Analysis

Descriptive statistics were used to summarize respondent characteristics and questionnaire answers. Categorical variables are presented as frequencies and percentages, *n* (%). Percentages were calculated using all respondents in the relevant survey as the denominator. Missing responses are reported separately. Comparisons between survey 1 and survey 2 were performed using the chi-square test or Fisher’s exact test, as appropriate.

For the 2025–2026 season, respondents were further stratified according to willingness to administer nirsevimab. Parents who were not in favor or were unsure were compared with parents who were in favor of nirsevimab administration. Differences between groups were assessed using chi-square or Fisher’s exact tests for categorical variables.

For the analysis according to month of birth, acceptance rates were compared between September–December and January–March within each season, and between the corresponding periods across the two seasons. A *p*-value < 0.05 was considered statistically significant. No formal a priori sample-size calculation was performed.

The study used a pragmatic recruitment approach in which all eligible parents or legal guardians encountered at the participating centers during the predefined survey periods were invited to participate. The final sample size therefore reflected the number of eligible individuals approached during each study period who agreed to complete the questionnaire. Given the exploratory and descriptive nature of the surveys, the objective was to maximize recruitment during each season rather than to recruit a predetermined number of participants.

## 3. Results

Table 1 shows the sociodemographic characteristics of respondents in the two surveys.

The two cohorts were broadly comparable with respect to parental age and family composition. The overall distribution of educational qualifications differed between the two surveys. The proportion of respondents with secondary-school education decreased from 15.74% to 10.27%, whereas the proportions with a diploma or university degree increased modestly from 39.73% to 43.37% and from 40.31% to 43.94%, respectively. Thus, the observed change reflected a modest shift toward higher educational qualifications rather than a downward shift. A significant difference emerged in neonatal comorbidities. The proportion of neonates with underlying comorbidities was markedly lower in survey 2 than in survey 1, decreasing from 16.79% to 5.19% (*p* < 0.001). Consistently, prematurity was significantly less frequent in survey 2, reported in 2.88% of neonates compared with 14.01% in survey 1 (*p* < 0.001). Other comorbidities were reported in 1.34% of neonates in survey 1 and 2.30% in survey 2, with no significant difference between the two groups.

Table 2 summarizes knowledge and perception about RSV and nirsevimab in the two surveys.

Compared with Survey 1, Survey 2 respondents more frequently reported awareness of RSV-related risks and more frequently correctly identified nirsevimab as an antibody. In contrast, previous awareness of nirsevimab was lower in Survey 2.

Table 3 describes the willingness to administer nirsevimab to the child, familiarity with nirsevimab, and trust in healthcare in the two surveys.

Overall willingness to administer nirsevimab remained high and similar between seasons. The most relevant changes concerned the factors influencing parental decision-making and perceptions regarding safety, whereas trust in the national healthcare system and willingness to pay remained broadly stable.

Willingness to receive nirsevimab was analyzed according to the neonate’s month of birth and compared between the 2024–2025 and 2025–2026 seasons (Table 4).

In 2024–2025, acceptance remained stable throughout the campaign, with similar rates in November–December and January–March (88.3% vs. 87.9%; *p* = 0.836). In contrast, during 2025–2026, acceptance was significantly higher among infants born in September–December than among those born in January–March (95.3% vs. 79.4%; *p* < 0.001), with a progressive decline from 82.6% in January to 76.5% in March. Compared with the previous season, acceptance was higher in September–December (95.3% vs. 88.3%; *p* < 0.01) but lower in January–March (79.4% vs. 87.9%; *p* < 0.001). Overall, these findings indicate a marked late-season decline in parental willingness during the 2025–2026 campaign.

Table 5 shows the variables associated with willingness to administer nirsevimab in the season 2025–2026.

In Survey 2, acceptance of nirsevimab was associated with several parental characteristics and perceptions. Parents in favor were more likely to have a university degree, be aware of RSV-related risks, perceive their child as being at greater risk of infection, have previous knowledge of nirsevimab, and correctly identify it as an antibody. They also reported greater confidence in nirsevimab effectiveness, lower concern about adverse effects, and greater trust in both pediatricians and the national healthcare system. Acceptance was additionally associated with greater interest in receiving information and with willingness to pay approximately EUR 250 if nirsevimab were not publicly funded. In contrast, parental age was not significantly associated with acceptance.

## 4. Discussion

This study provides an updated evaluation of parental knowledge, perceptions, and willingness to accept nirsevimab for RSV prevention across two consecutive RSV seasons in Emilia-Romagna, Italy. Overall, willingness to administer nirsevimab remained very high and stable, increasing slightly from 87.0% in the 2024–2025 season to 88.0% in the 2025–2026 season. This finding is reassuring, particularly because the second survey was conducted after the first regional implementation season and after the prophylaxis strategy had been expanded. The persistence of high acceptance suggests that nirsevimab was generally well received by families and that no major decline in parental confidence occurred after its initial introduction.

An important consideration is that the primary outcome of this study was parental willingness to have nirsevimab administered rather than verified administration of nirsevimab. Actual receipt of prophylaxis represents the definitive behavioral outcome and is clearly more relevant for evaluating program coverage and effectiveness. However, the objective of the present survey was different: it was specifically designed to investigate parental decision-making at the time of counseling and to identify potentially modifiable determinants of acceptance or hesitancy, including knowledge of RSV, understanding of nirsevimab, perceived efficacy and safety, trust in healthcare professionals, and economic considerations. These factors cannot be adequately characterized from administration records alone, because non-administration may result not only from parental refusal but also from eligibility, timing, availability, organization of care, or other logistical factors.

Assessment of willingness therefore provides information on an intermediate but clinically relevant stage in the pathway between communication and actual uptake. In particular, it enables identification of misconceptions, concerns, and information needs before the final decision is implemented and may consequently inform communication strategies aimed at improving acceptance. Nevertheless, willingness should not be considered equivalent to actual administration, and the present results cannot be interpreted as estimates of nirsevimab coverage.

We agree that linking parental intentions with subsequent verified administration would provide a stronger assessment of whether stated acceptance translates into behavior. Such linkage was not possible in the present study because questionnaires were collected anonymously and were not linked to individual clinical or immunization records. Future studies should prospectively combine parental attitude surveys with verified administration data to evaluate the intention–behavior relationship and determine which factors predict actual uptake.

The high level of acceptance observed in our study is consistent with early European experiences. In France, a prospective maternity-based study conducted during the first season of nirsevimab implementation reported high parental acceptance and reassuring short-term safety findings [19]. Similarly, in Galicia, Spain, the universal nirsevimab program achieved coverage exceeding 90%, supporting the feasibility of broad infant immunization when prophylaxis is actively integrated into regional public health programs [20]. The Spanish NIRSE-GAL study also showed substantial real-world impact on RSV-related hospitalization, further strengthening the rationale for universal or expanded prophylaxis strategies [20,21].

In contrast, experiences from the United States have shown more variable uptake. A pediatric primary care network in Washington, DC, reported that only 38.4% of eligible infants received nirsevimab during the 2023–2024 RSV season, a much lower proportion than that observed in our setting [22]. This difference likely reflects contextual factors, including product availability, delivery setting, reimbursement mechanisms, timing of administration, and organization of the immunization pathway. In the United States, uptake during the first season was also affected by supply limitations and implementation barriers, whereas in Emilia-Romagna nirsevimab was offered within a publicly organized regional campaign. These differences highlight that parental willingness alone is not sufficient to ensure high coverage; programmatic accessibility, clear delivery pathways, and sustained communication throughout the entire RSV season are equally important [22,23].

One of the most relevant findings of the present study comes from the analysis according to month of birth. During the 2024–2025 season, willingness to receive nirsevimab remained stable across the whole observation period, with no significant difference between infants born in November–December and those born in January–March. In contrast, a clear decline emerged during the 2025–2026 season. Acceptance was very high among infants born between September and December, reaching 95.3% overall, but decreased significantly among those born between January and March, when overall acceptance fell to 79.4% (*p* < 0.001). Moreover, acceptance declined progressively across the final months of the season, from 82.6% in January to 79.1% in February and 76.5% in March. Compared with the previous season, willingness among infants born in January–March decreased significantly, from 87.9% in 2024–2025 to 79.4% in 2025–2026 (*p* < 0.001).

This seasonal decline is clinically and programmatically important. Although overall acceptance remained high, the reduction observed in the final part of the 2025–2026 season suggests that parents may perceive nirsevimab as less necessary when the RSV season is considered to be approaching its end. This perception may be reinforced by the fact that RSV is commonly understood as a winter infection, leading some families to underestimate the potential benefit of prophylaxis for infants born later in the season. However, such a perception may be misleading. RSV circulation can persist into late winter and early spring, and seasonality has become less predictable after the COVID-19 pandemic, with earlier, delayed, shorter, or atypical epidemic patterns reported in several countries [24]. Therefore, infants born in the final months of the campaign may still be exposed to RSV during a vulnerable period of life and should not be excluded, directly or indirectly, from the benefits of prevention.

Maintaining high coverage throughout the entire RSV season is therefore essential. The effectiveness of a universal or expanded nirsevimab program depends not only on high uptake at the beginning of the campaign, but also on the capacity to sustain acceptance among infants born later. A decline in late-season administration could create a subgroup of unprotected infants who remain susceptible during ongoing RSV circulation or during possible shifts in epidemic timing. This is particularly relevant because the highest risk of severe RSV disease is concentrated in the first months of life, regardless of whether birth occurs at the beginning or toward the end of the traditional RSV season [24,25]. From a public health perspective, reduced late-season coverage could weaken the overall impact of the program on hospitalizations and healthcare burden, even when early-season uptake is excellent.

These findings suggest that communication strategies should be adapted across the season. At the beginning of the campaign, messages may focus on the start of RSV circulation and the importance of early protection. In the final months, however, counseling should explicitly address the misconception that prophylaxis is no longer useful. Parents of infants born in January, February, and March should receive clear information that RSV may still circulate, that newborns remain highly vulnerable, and that nirsevimab can still provide meaningful protection during the remaining period of exposure. This message should be consistently delivered by neonatologists, maternity staff, primary care pediatricians, and public health professionals. Campaign fatigue among healthcare workers should also be considered, as the intensity of counseling may decline after the initial months of implementation. Standardized information materials and reminders could help maintain the same level of recommendation throughout the season.

An additional finding of our study is that awareness of RSV-related risks increased between the two surveys. The proportion of parents who reported being aware of RSV risks rose from 68.2% to 73.5%, and the proportion who reported not knowing the main risks associated with RSV decreased markedly. This suggests that public and professional communication about RSV may have improved after the first season of nirsevimab use. However, despite this overall improvement, some specific RSV-related complications were identified less frequently in the second survey, including bronchiolitis, pneumonia, hospitalization for respiratory failure, and bronchospasm. This apparently contradictory finding may reflect differences in the respondent population, changes in counseling exposure, or a shift from detailed disease knowledge toward more general awareness of RSV as a relevant infant infection. It may also indicate that parents increasingly recognize RSV as important but do not always retain specific clinical information about its complications.

The need for accurate information remains central. RSV is a major cause of lower respiratory tract infection and hospitalization in infants, and severe disease can occur also in healthy term infants, not only in those with recognized risk factors [24,25]. This point is particularly relevant for communication campaigns, because parents may underestimate the need for prophylaxis if they perceive RSV as dangerous only for premature infants or children with comorbidities. In our study, acceptance was strongly associated with perceiving the child as being at moderate or high risk of RSV infection, confirming that perceived susceptibility remains a key driver of preventive behavior. This is particularly relevant in the final part of the season, when perceived susceptibility may decline despite the persistence of potential exposure.

Knowledge of nirsevimab also evolved between seasons. Although previous awareness of nirsevimab was lower in survey 2 than in survey 1, the proportion of respondents correctly identifying nirsevimab as an antibody that helps prevent infections increased from 65.9% to 71.7%. This is encouraging because misunderstanding nirsevimab as a vaccine remains common and may influence parental attitudes. Similar difficulties have been described in other studies evaluating parental perceptions of new RSV preventive products, where monoclonal antibodies were often conceptually grouped with vaccines or other medications [26,27]. Clear explanation of the mechanism of action of nirsevimab is therefore important, particularly because parents may transfer vaccine-related hesitancy or misconceptions to monoclonal antibody prophylaxis if the distinction is not adequately explained.

An unexpected finding was the lower proportion of parents reporting previous awareness of nirsevimab in Survey 2. The item referred to awareness before the counseling encounter. Thus, the result does not indicate that parents remained uninformed after receiving counseling within the regional program. The earlier and broader second-season campaign may have included a less selected population of parents presenting soon after delivery, before contact with a primary care pediatrician or exposure to campaign information. Differences in recruitment timing and cohort composition may therefore have contributed. Because individual exposure to the regional communication campaign was not measured, the finding should be interpreted cautiously and cannot be considered evidence that regional awareness declined.

The role of healthcare professionals remains one of the most relevant determinants of acceptance. In the 2025–2026 survey, parents in favor of nirsevimab were significantly more likely to report high trust in their pediatrician and in the national healthcare system. They were also more likely to indicate the pediatrician’s recommendation as an important factor influencing their decision. These findings are consistent with the broader literature on immunization decision-making, which consistently identifies healthcare provider recommendation as one of the strongest predictors of uptake [23,28]. The CDC has similarly emphasized that clinicians play a critical role in improving RSV immunization uptake through strong recommendations and clear communication on safety and effectiveness [23].

Interestingly, the reported influence of the primary care pediatrician decreased from 71.2% in survey 1 to 48.7% in survey 2. This does not necessarily indicate reduced trust, because the proportion of parents reporting very high trust in their pediatrician remained stable across seasons. Rather, it may reflect the different organization of the second campaign, which started earlier and was expanded to all infants, potentially increasing the role of hospital-based or public health communication. It may also reflect the higher proportion of respondents in survey 2 who did not yet have a pediatrician. This finding has practical implications: counseling should not rely exclusively on the primary care pediatrician, especially when decisions must be made very early in life or before formal pediatric assignment. Neonatologists, maternity staff, public health services, and primary care pediatricians should deliver consistent and coordinated messages from the beginning to the end of the RSV season.

Safety concerns remained an important component of parental decision-making. In both surveys, approximately one in ten respondents reported being very concerned about possible side effects. However, moderate concern decreased and the proportion reporting only little concern increased in survey 2. Parents in favor of nirsevimab were much less likely to be very concerned about side effects than those not in favor or unsure. This pattern is consistent with previous studies showing that safety concerns are among the main reasons for hesitancy toward newly introduced RSV preventive products [19,23,26]. It is noteworthy that many respondents were unable to identify specific adverse events, suggesting that concern may often be non-specific and related to the novelty of the product rather than to defined safety signals. Communication should therefore provide balanced information: acknowledging the possibility of mild local or systemic reactions while emphasizing the reassuring safety profile reported in clinical trials and real-world studies [19,29].

The perceived efficacy of nirsevimab was also associated with acceptance. In survey 2, almost half of parents in favor considered nirsevimab very effective, compared with only 12.4% of those not in favor or unsure. Conversely, uncertainty about efficacy was very common among hesitant parents. These results align with evidence from implementation studies showing that confidence in effectiveness is a key determinant of uptake [23,26]. They are also supported by growing real-world evidence demonstrating that nirsevimab substantially reduces RSV-related hospitalization and severe outcomes in infants [20,21,29]. In this context, communicating real-world effectiveness data may be particularly useful, because parents may find population-level reductions in hospitalizations more understandable and persuasive than trial efficacy estimates alone. Such communication should be maintained throughout the season, not only during campaign launch.

Economic considerations also emerged as important. Although willingness to pay approximately EUR 250 remained stable across the two surveys, acceptance was much higher among parents already in favor of nirsevimab than among those not in favor or unsure. This finding confirms that out-of-pocket costs could amplify hesitancy and reduce equitable access. International implementation experience supports this concern: financial burden, purchasing costs, and reimbursement issues have been identified as major barriers to RSV immunization delivery, especially in systems where providers or families face upfront costs [23]. In publicly funded settings, removing direct costs for families is likely to be essential to maintain high uptake and avoid socioeconomic disparities. Although willingness to pay was more frequent among parents in favor of nirsevimab, the study did not assess its relationship with educational level, and household income was not collected. Educational attainment should not be considered a direct proxy for income. Willingness to pay may reflect a combination of perceived value, confidence in the intervention, concern about RSV, and financial capacity; these components could not be distinguished in the present study.

The comparison between parents in favor of nirsevimab and those not in favor or unsure confirms several determinants of acceptance. Higher educational level, awareness of RSV risks, correct identification of nirsevimab as an antibody, perceived child susceptibility, confidence in efficacy, lower concern about adverse events, trust in pediatricians, trust in the healthcare system, and willingness to pay were all associated with favorable attitudes. These findings are consistent with previous literature on parental acceptance of infant RSV prevention, in which knowledge, perceived disease severity, confidence in healthcare providers, and safety perceptions are recurrent determinants [28,29,30]. They also suggest that hesitancy toward nirsevimab in our population is not primarily driven by generalized opposition to preventive medicine, but rather by modifiable factors such as uncertainty, limited knowledge, insufficient trust, and possibly reduced perceived need later in the season.

This study has several strengths. It includes two large samples collected across consecutive seasons in the same Italian region, allowing comparison before and after the first implementation year. The use of a similar questionnaire across surveys enabled evaluation of changes over time in parental perceptions and willingness. The study also assessed several dimensions of decision-making, including disease awareness, product knowledge, perceived efficacy and safety, trust, information preferences, willingness to pay, and seasonal timing of acceptance.

Some limitations should also be acknowledged. First, the study assessed self-reported willingness and perceptions, which may not always correspond to actual administration or verified coverage. Therefore, the reduction observed in the final part of the season should be interpreted as a decline in reported acceptance or willingness, although it may plausibly translate into lower real-world uptake. Although intention is informative for understanding parental decision-making, it may overestimate or underestimate subsequent behavior. Because questionnaires were anonymous, we could not determine whether individual parents who expressed willingness ultimately had nirsevimab administered to their child. Second, the two survey populations were not identical, and differences in neonatal comorbidities, timing of recruitment, and campaign organization may have influenced responses. Third, social desirability bias may have led some parents to overstate their willingness to accept nirsevimab. Fourth, the study was conducted in Emilia-Romagna, a region with a specific healthcare organization and public health strategy, and the findings may not be fully generalizable to other Italian regions or countries with different implementation models. Fifth, because the total number of eligible families approached was not systematically recorded, survey-level response and non-response rates could not be calculated. This limits assessment of potential non-response bias, although item-level missing responses are reported throughout the tables. Sixth, the two cohorts differed in neonatal clinical characteristics, with lower reported comorbidity and prematurity in Survey 2. This finding should not be interpreted as a temporal reduction in the regional prevalence of these conditions. The surveys comprised independent samples rather than repeated observations of the same population. Moreover, the second campaign started earlier and had broader eligibility, which may have increased the representation of healthy term infants. Differences in recruitment timing, participating clinical pathways, and the relative contribution of maternity and neonatal settings may also have affected cohort composition. As these mechanisms were not directly assessed, residual selection bias cannot be excluded. Seventh, the analyses of factors associated with acceptance were univariable and do not establish independent associations. Because several respondent characteristics and attitudes may be interrelated, residual confounding cannot be excluded. An additional limitation is the absence of a multivariable analysis. The associations identified in the present study were based on univariate comparisons and therefore do not allow determination of the independent contribution of individual factors to parental acceptance of nirsevimab. Potential confounding between sociodemographic characteristics, knowledge of RSV and nirsevimab, perceived susceptibility, safety concerns, and trust in healthcare professionals cannot therefore be excluded. Future studies should incorporate multivariable regression models to identify independent predictors of acceptance and better characterize the relative contribution of these factors to parental decision-making. Finally, although the survey provides valuable quantitative data, qualitative research could further clarify the reasons behind persistent uncertainty, refusal, and the decline in acceptance observed among infants born later in the RSV season.

Several methodological measures were adopted to reduce potential sources of bias. First, both surveys were conducted in multiple centers within the same region and used the same core questionnaire and a comparable recruitment procedure, thereby improving consistency between seasons. Second, questionnaire development included multidisciplinary expert review and pilot testing among parents with heterogeneous educational, linguistic, and health-literacy backgrounds, with wording subsequently refined to maximize comprehensibility. Third, participation was voluntary and questionnaires were anonymous, which was intended to reduce pressure to provide socially desirable responses. The questionnaire was administered in the context of routine counseling using a standardized structure, and the same principal response categories were retained across seasons to permit direct comparison. Finally, respondents were allowed to leave individual questions unanswered rather than being required to provide a response, thereby reducing forced or arbitrary answers.

Nevertheless, these measures cannot completely eliminate the limitations inherent in repeated cross-sectional questionnaire studies. The two survey populations were independent and not identical, and differences in recruitment timing, campaign eligibility, neonatal characteristics, and exposure to information may have influenced between-season comparisons. Social-desirability and selection biases also remain possible, and the findings may not be fully generalizable outside Emilia-Romagna.

## 5. Conclusions

This updated survey shows that parental willingness to administer nirsevimab remained high across two consecutive RSV seasons in Emilia-Romagna. However, the marked decline in acceptance among infants born between January and March during the 2025–2026 season represents an important signal for future campaigns. RSV prevention programs should not focus exclusively on achieving high uptake at the beginning of the season, but should also ensure that coverage remains high until the end of the campaign. Sustained communication, repeated counseling, and consistent recommendations from all healthcare professionals are needed to prevent late-season declines in acceptance and to ensure that infants born in the final part of the RSV season receive the same opportunity for protection as those born earlier.

## Figures and Tables

**Table 1:** Self-reported sociodemographic characteristics of respondents in survey 1 and survey 2.

	Survey 1 (2024–2025) N = 1042		Survey 2 (2025–2026) N = 867		*p*-Value
	*n*	%	*n*	%	
Parent(s) who filled in the questionnaire					
Father	332	31.86	267	30.80	0.326
Mother	696	66.79	572	65.97	0.371
Both	10	0.96	28	3.23	<0.001
Missing	4	0.38			
Age of the parent					
≤20 years	4	0.96	8	0.92	0.117
>20–≤30 years	261	25.05	199	22.95	0.287
>30–≤40 years	661	63.44	554	63.90	0.834
>40 years	109	10.46	106	12.23	0.224
Missing	1	0.10			
Education					
First-grade school	13	1.25	7	0.81	0.347
Secondary school	164	15.74	89	10.27	<0.001
Diploma	414	39.73	376	43.37	0.108
Degree	420	40.31	381	43.94	0.109
Missing	31	2.98	14	1.61	0.051
Number of children in the family					
First child	540	51.82	434	50.06	0.442
Second child	380	36.47	315	36.33	0.951
≥Third child	122	11.71	118	13.61	0.212
Underlying comorbidities in the neonate					<0.001
None	862	82.73	814	93.89	
Yes	175	16.79	45	5.19	
Missing	5	0.48	8	0.92	
Type of comorbidity in the neonate					
Prematurity	146	14.01	25	2.88	<0.001
Other	14	1.34	20	2.30	0.113

**Table 2:** Knowledge and perception about RSV and nirsevimab in survey 1 and survey 2.

	Survey 1 (2024–2025) N = 1042		Survey 2 (2025–2026) N = 867		*p*-Value
	*n*	%	*n*	%	
Are you aware of the risks associated with RSV					<0.05
No	315	30.23	218	25.14	
Yes	711	68.23	637	73.47	
Missing			12	1.38	
How much do you think your child is at risk of RSV infection?					
Not at all	68	6.53	47	5.42	0.312
A little	266	25.53	222	25.61	0.969
Moderately	506	48.56	446	51.44	0.210
Very much	162	15.55	123	14.19	0.406
Missing	40	3.84	29	3.34	0.565
What do you think are the main risks associated with RSV?					
I don’t know	170	16.31	48	5.54	<0.001
No risk	20	1.92	5	0.58	<0.05
Bronchiolitis	749	71.88	335	38.64	<0.001
Pneumonia	200	19.19	95	10.96	<0.001
Hospitalization for respiratory failure	485	46.55	207	23.88	<0.001
Hospitalization in intensive care unit	268	25.72	253	29.18	0.091
Bronchospasm	148	14.20	77	8.88	<0.001
Asthma	54	5.18	75	8.65	<0.05
Missing			11	1.27	<0.001
Have you ever heard of nirsevimab before?					<0.001
No	344	33.01	568	65.51	
Yes	689	66.12	294	33.91	
Missing	9	0.86	5	0.58	
If yes, where did you get information about nirsevimab?					
Internet/TV/Social	109	31.69	58	6.69	<0.001
Primary care pediatrician	103	29.94	50	5.77	<0.01
Neonatologist	52	15.12	44	5.07	0.933
Gynecologist	44	12.79	24	2.77	0.088
Friends	64	18.60	22	2.54	<0.001
Other	55	15.99	19	2.19	<0.001
Hospital	8	2.33	16	1.85	<0.05
Prenatal class	11	3.20	5	0.58	0.187
University	3	0.87	2	0.23	0.586
What do you think nirsevimab is?					
A vaccine	243	23.32	198	22.84	0.803
An antibody that helps prevent infections	687	65.93	622	71.74	<0.01
A regular medication	5	3.36	21	2.42	<0.001

**Table 3:** Willingness to administer nirsevimab to the child, familiarity with nirsevimab, and trust in healthcare in survey 1 and survey 2.

	Survey 1 (2024–2025) N = 1042		Survey 2 (2025–2026) N = 867		*p*-Value
	*n*	%	*n*	%	
Are you willing to have nirsevimab administered to your child?					
No	23	2.21	33	3.81	<0.05
Yes	907	87.04	763	88.00	0.528
Not sure	92	8.83	56	6.46	0.054
missing			15	1.73	
What factors most influence your decision to have nirsevimab administered to your child?					
Primary care pediatrician’s recommendation	742	71.21	422	48.67	<0.001
Opinion of friends	59	5.66	20	2.31	<0.001
Information from media sources	65	6.24	15	1.73	<0.001
Safety data	456	43.76	167	19.26	<0.001
Efficacy data	409	39.25	159	18.34	<0.001
Other	59	5.66	11	1.27	<0.001
How effective do you believe nirsevimab is in protecting your child from RSV?					
I don’t know	381	36.56	279	32.18	<0.05
Very much	441	42.32	384	44.29	0.387
Moderately	203	19.48	176	20.30	0.655
A little	3	0.29	17	1.96	<0.001
Not at all	2	0.19	-	-	0.197
missing	12	1.15	11	1.27	0.815
How concerned are you about the possible side effects of nirsevimab?					
Very much	111	10.65	103	11.88	0.397
Moderately	412	39.54	291	33.56	<0.01
A little	374	35.89	358	41.29	<0.05
Not at all	130	12.48	99	11.42	0.479
Missing	15	1.44	16	1.85	0.485
What do you think might be the side effects of nirsevimab?					
I don’t know	354	33.97	304	35.06	0.618
Fever	608	58.35	451	52.02	<0.01
Pain	286	27.45	106	12.23	<0.001
Skin rash	286	27.45	110	12.69	<0.001
Other	32	3.07	3	0.35	<0.001
How important is it for you to receive detailed information about the side effects and risks associated with nirsevimab?					
Very important	866	83.11	684	78.89	<0.05
Moderately important	131	12.57	138	15.92	<0.05
A little important	29	2.78	25	2.88	0.895
Not important	8	0.77	5	0.58	0.613
Missing	8	0.77	15	1.73	0.055
How much do you trust your pediatrician when it comes to advice about your child’s health?					
I don’t have a pediatrician yet	195	18.71	206	23.76	<0.01
Very much	663	63.63	547	63.09	0.809
Moderately	160	15.36	90	10.38	<0.01
A little	10	0.96	13	1.50	0.282
Not at all	2	0.19	2	0.23	0.854
Missing	12	1.15	9	1.04	0.813
Would you be interested in receiving more information about nirsevimab and its indications?					0.054
Yes	869	83.40	697	80.39	
No	152	14.59	155	17.88	
Missing	21	2.02	15	1.73	
How would you prefer to receive information about nirsevimab?					
Conversation with a neonatologist	270	25.91	237	27.34	0.483
Conversation with primary care pediatrician	518	49.71	423	48.79	0.688
Conversation with a trusted doctor	93	8.93	75	8.65	0.833
Informational material	272	26.10	232	26.76	0.746
Internet/social media	104	9.98	66	7.61	0.070
Other	40	3.84	9	1.04	<0.001
How much do you trust the national healthcare system regarding the safety and effectiveness of nirsevimab?					
Very much	591	56.72	499	57.55	0.713
Moderately	396	38.00	310	35.76	0.311
A little	38	3.65	41	4.73	0.237
Not at all	5	0.48	4	0.46	0.953
Missing	12	1.15	13	1.50	0.506
If the cost of nirsevimab were the family’s responsibility and the medication cost approximately EUR 250, would you be willing to pay?					0.800
Yes	702	67.37	572	65.97	
No	311	29.85	260	29.99	
Missing	29	2.78	35	4.04	

**Table 4:** Willingness to receive nirsevimab according to month of birth.

	Season 2024–2025					Season 2025–2026				
	Born in the Period	Yes	%	No	%	Born in the Period	Yes	%	No	%
September	NA	NA	NA	NA	NA	114	110	96.5	4	3.5
October	NA	NA	NA	NA	NA	114	109	95.6	5	4.4
November	217	191	88.0	26	12	117	112	95.7	5	4.3
December	219	194	88.6	25	11.4	124	116	93.5	8	6.5
**Total Sept–Dec**	**436**	**385**	**88.3**	**51**	**11.7**	**469**	**447**	**95.3**	**22**	**4.7**
January	224	196	87.5	28	12.5	132	109	82.6	23	17.4
February	212	188	88.7	24	11.3	134	106	79.1	28	20.9
March	158	138	87.3	20	12.7	132	101	76.5	31	23.5
**Total Jan–Mar**	**594**	**522**	**87.9**	**72**	**12.1**	**398**	**316**	**79.4**	**82**	**20.6**

NA, not available. Chi-square test results: Jan–Mar vs. Sept–Dec (Session 2024–2025): *p*-value = 0.836; Jan–Mar vs. Sept–Dec (Session 2025–2026): *p*-value < 0.001; Sept–Dec Session 2024–2025 vs. Sept–Dec Session 2025–2026: *p*-value < 0.01; Jan–Mar Session 2024–2025 vs. Jan–Mar Session 2025–2026: *p*-value < 0.001.

**Table 5:** Variables that showed significant differences between those who were not in favor or were unsure about having nirsevimab administered to their child and those who were in favor.

	Not in Favor or Unsure of Nirsevimab (*n* = 89)		In Favor of Nisevimab (*n* = 763)		*p*-Value
Age of the parent					0.437
≤20 years	2	2.25	6	0.79	
>20–≤30 years	22	24.72	173	22.67	
>30–≤40 years	54	60.67	489	64.09	
>40 years	11	12.36	95	12.45	
Education					<0.001
First-grade school	2	2.25	5	0.66	
Secondary school	12	13.48	74	9.70	
Diploma	49	55.06	320	41.94	
Degree	20	22.47	360	47.18	
Missing	6	6.74	4	0.52	
Are you aware of the risks associated with RSV?					<0.001
No	43	48.31	173	22.67	
Yes	44	49.44	589	77.20	
How much do you think your child is at risk of RSV infection?					<0.001
Not at all	10	11.24	36	4.72	
A little	36	40.45	186	24.38	
Moderately	35	39.33	408	53.47	
Very	4	4.49	118	15.47	
Missing	4	4.49	15	1.97	
What do you think are the main risks associated with RSV?					
I don’t know	10	11.24	36	4.72	<0.05
No risk			5	0.66	0.574
Bronchiolitis	20	22.47	311	40.76	<0.001
Pneumonia	8	8.99	85	11.14	0.338
Hospitalization for respiratory failure	11	12.36	194	25.43	<0.01
Hospitalization in intensive care unit	8	8.99	245	32.11	<0.001
Bronchospasm	4	4.49	72	9.44	0.079
Asthma	3	3.37	71	9.31	<0.05
Missing			3	0.39	0.718
Have you ever heard of nirsevimab before?					<0.001
No	72	80.90	487	63.83	
Yes	16	17.98	276	36.17	
missing	1	1.12			
What do you think nirsevimab is?					
A vaccine	31	34.83	165	21.63	<0.01
An antibody that helps prevent infections	49	55.06	568	74.44	<0.001
A regular medication	6	6.74	15	1.97	<0.05
What factors most influence your decision to have nirsevimab administered to your child?					
Primary care pediatrician’s recommendation	30	33.71	390	51.11	<0.01
Opinion of friends	3	3.37	17	2.23	0.348
Information from media sources	1	1.12	14	1.83	0.524
Safety data	15	16.85	150	19.66	0.318
Efficacy data	8	8.99	150	19.66	<0.01
Other	2	2.25	9	1.18	0.322
How effective do you believe nirsevimab is in protecting your child from RSV?					<0.001
I don’t know	64	71.91	210	27.52	
Very much	11	12.36	371	48.62	
Moderately	12	13.48	164	21.49	
A little	2	2.25	15	1.97	
Not at all					
missing			3	0.39	
How concerned are you about the possible side effects of nirsevimab?					<0.001
Very much	36	40.45	65	8.52	
Moderately	34	38.20	255	33.42	
A little	14	15.73	343	44.95	
Not at all	3	3.37	95	12.45	
Missing	2	2.25	5	0.66	
What do you think might be the side effects of nirsevimab?					
I don’t know					
Fever	26	29.21	419	54.91	<0.001
Pain	7	7.87	97	12.71	0.122
Skin rash	5	5.62	102	13.37	<0.05
Other			3	0.39	0.718
How important is it for you to receive detailed information about the side effects and risks associated with nirsevimab?					<0.05
Very important	63	70.79	615	80.60	
Moderately important	16	17.98	122	15.99	
A little important	3	3.37	22	2.88	
Not important	3	3.37	2	0.26	
Missing	4	4.49	2	0.26	
How much do you trust your pediatrician when it comes to advice about your child’s health?					<0.001
I don’t have a pediatrician yet	29	32.58	174	22.80	
Very much	38	42.70	505	66.19	
Moderately	18	20.22	72	9.44	
A little	4	4.49	9	1.18	
Not at all			2	0.26	
Missing			1	0.13	
Would you be interested in receiving more information about nirsevimab and its indications?					<0.05
Yes	64	71.91	628	82.31	
No	23	25.84	131	17.17	
Missing	2	2.25	4	0.52	
How would you prefer to receive information about nirsevimab?					
Conversation with a neonatologist	20	22.47	216	28.31	0.149
Conversation with primary care pediatrician	47	52.81	370	48.49	0.255
Conversation with a trusted doctor	12	13.48	63	8.26	0.079
Informational material	14	15.73	218	28.57	<0.01
Internet/social media	6	6.74	60	7.86	0.453
Other					
How much do you trust the national healthcare system regarding the safety and effectiveness of nirsevimab?					<0.001
Very much	25	28.09	470	61.60	
Moderately	48	53.93	260	34.08	
A little	13	14.61	28	3.67	
Not at all	3	3.37	1	0.13	
Missing					
If the cost of nirsevimab were the family’s responsibility and the medication cost approximately EUR 250, would you be willing to pay?					
Yes	33	37.08	536	70.25	<0.001
No	53	59.55	205	26.87	
Missing	3	3.37	22	2.88	

## Data Availability

All the available data are included in the manuscript.
